# Outcomes of postnatal systemic corticosteroids administration in ventilated preterm newborns: a systematic review of randomized controlled trials

**DOI:** 10.3389/fped.2024.1344337

**Published:** 2024-02-14

**Authors:** Giovanni Boscarino, Viviana Cardilli, Maria Giulia Conti, Federica Liguori, Paola Repole, Pasquale Parisi, Gianluca Terrin

**Affiliations:** ^1^ Department of Maternal and Child Health, Policlinico Umberto I, Sapienza University of Rome, Rome, Italy; ^2^ Pediatrics Unit, Department of Neuroscience, Mental Health and Sense Organs (NESMOS), Faculty of Medicine and Psychology, Sant’ Andrea Hospital, Sapienza University of Rome, Rome, Italy

**Keywords:** hydrocortisone, dexamethasone, neurodevelopment, growth, extubation, pulmonary outcome, systemic hypertension, hyperglycemia

## Abstract

**Introduction:**

Prolonged mechanical ventilation, commonly used to assist preterm newborns, increases the risk of developing bronchopulmonary dysplasia (BPD). In recent decades, studies have demonstrated that systemic corticosteroids play a significant role in the prevention and management of BPD. In this systematic review of randomized controlled trials (RCTs), we evaluated the association between the administration of systemic corticosteroids in preterm infants and its long-term outcomes, such as neurodevelopment, growth, extubation rate, and related adverse effects.

**Methods:**

We conducted an electronic search in Medline, Scopus, and PubMed using the following terms: “premature infants” and “corticosteroids.” We considered all RCTs published up to June 2023 as eligible. We included all studies involving preterm newborns treated with systemic corticosteroids and excluded studies on inhaled corticosteroids.

**Results:**

A total of 39 RCTs were evaluated. The influence of steroids administered systemically during the neonatal period on long-term neurological outcomes remains unknown, with no influence observed for long-term growth. The postnatal administration of systemic corticosteroids has been found to reduce the timing of extubation and improve respiratory outcomes. Dexamethasone appears to be more effective than hydrocortisone, despite causing a higher rate of systemic hypertension and hyperglycemia. However, in the majority of RCTs analyzed, there were no differences in the adverse effects related to postnatal corticosteroid administration.

**Conclusion:**

Dexamethasone administered during the neonatal period appears to be more effective than hydrocortisone in terms of respiratory outcomes; however, caution should be taken when administering dexamethasone. Data derived from current evidence, including meta-analyses, are inconclusive on the long-term effects of the administration of systemic steroids in preterm infants or the possibility of neurodevelopmental consequences.

## Introduction

The survival rate of preterm newborns has improved over the last 20 years due to advances in neonatal care (1). However, the improvement in survival has been associated with an increased morbidity rate and reduced long-term neurodevelopmental (NDV) outcomes (2, 3). Two of the most important innovations in neonatal care are the introduction of surfactant therapy and the improvement in mechanical ventilation (MV). However, prolonged MV (PMV) is harmful and increases the risk of developing bronchopulmonary dysplasia (BPD) (4, 5). BPD is a chronic inflammatory lung disease of premature neonates characterized by impaired lung development (4, 6). It has a multifactorial pathogenesis, wherein prolonged oxygen exposure induces a destructive local inflammatory response in the lung alveoli, associated with a simultaneous impaired repair response (6). In addition, it has been demonstrated that BPD is associated with impaired long-term NDV and pulmonary function outcomes (7, 8). Thus, neonatologists aim to extubate preterms as soon as possible, albeit not always possible, especially for extremely low gestational age newborns (ELGAN). Corticosteroids (e.g., dexamethasone and hydrocortisone) are currently administered intravenously (IV) or orally (PO) for the treatment and prevention of BPD. Studies have related their beneficial effects to their anti-inflammatory activity (9–11). The authors researched inhaled corticosteroids (12); however, no beneficial effects were found on the risk of neurological disability although the mortality rate was higher in the treated group, thus not allowing the routine administration (13, 14). However, whether the use of dexamethasone or hydrocortisone IV or PO improves or reduces long-term neurological outcomes is still debated. In addition, there are concerns regarding the prophylactic use of systemic corticosteroid therapy for possible adverse effects (i.e., sepsis, infection, and metabolic side effects).

In this systematic review of randomized controlled trials (RCTs), we studied the association between the administration of systemic corticosteroids during the neonatal period and its long-term outcomes, in terms of NDV and growth. Additionally, we evaluated the respiratory outcomes and possible adverse effects.

## Material and methods

### Studies, population, and intervention

We considered all RCTs published up to June 2023 as eligible. We included all studies involving preterm newborns treated with systemic (IV or PO) corticosteroids and excluded studies on inhaled corticosteroids.

### Outcomes

Our primary outcome was the long-term effects such as NDV and growth. Our secondary outcomes were as follows: the rate of extubation, reintubation, BPD, other respiratory outcomes (considering the duration of invasive or non-invasive MV, supplemental oxygen therapy, FiO2, or other specific ventilatory/respiratory data), and steroid-related adverse effects. We considered the following as adverse effects: systemic hypertension, hyperglycemia (HG), sepsis or other infections, patent ductus arteriosus (PDA), intraventricular hemorrhage (IVH), necrotizing enterocolitis (NEC), and retinopathy of prematurity (ROP).

### Research methods and study selection

We performed a systematic review of the published RCTs, in compliance with the Preferred Reporting Items for Systematic Reviews and Meta-analyses (PRISMA) guidelines (15). We conducted an electronic search in Medline, Scopus, and PubMed using the following medical subject headings and terms: “premature infants” and “corticosteroids.” Only English manuscripts and RCTs were considered. Two authors (G.B. and F.L.) independently assessed the study eligibility according to the pre-established criteria and performed an accurate check to exclude duplicates. A discussion with a third part researcher (G.T.) resolved different in opinion, to achieve consensus. We performed a manual search of the reference list of the systematic reviews and meta-analyses published and excluded them from this review.

### Data extraction, management, and risk of bias

Two authors (G.B. and F.L.) independently extracted the data from the selected articles using specifically designed data forms. For each selected RCT, the form summarized data on authorship, year of publication, population, inclusion and exclusion criteria, doses of steroids, days to extubation, duration of therapy, more than one cycle of steroids, and administration of other steroids. Another specific data form summarized the outcomes (e.g., extubation, reintubation, BPD, other respiratory outcomes, systemic hypertension, HG, sepsis/infection, PDA, IVH, NEC, ROP, and long-term outcomes). These data were checked for missing information, errors, and inconsistencies with published reports. If evidenced, differences were resolved by discussion and consensus between the researchers. The corresponding authors were contacted when the eligibility criteria of their papers were unclear.

The risk of bias was assessed independently by two researchers (G.B. and G.T.) using a specific form. We considered bias as selection bias (random sequence generation and allocation concealment), performance bias (blinding of the study personnel as to which intervention a neonate had received), detection bias (blinding of personnel evaluating outcomes), attrition bias (completeness of reporting data, reason, and balance across groups of missing data), reporting bias (reporting of the study's prespecified or expected outcomes of interest to the review), and other sources of bias (early interruption of the trial due to data-dependent process or bias related to the specific study design). We categorized the risks of bias as high, low, or unclear for each study, using standard methods (15). The selection bias was judged as unclear when these aspects were not available. The differences in opinion were resolved by discussion and consensus.

## Results

### Study description

**Figure 1:**
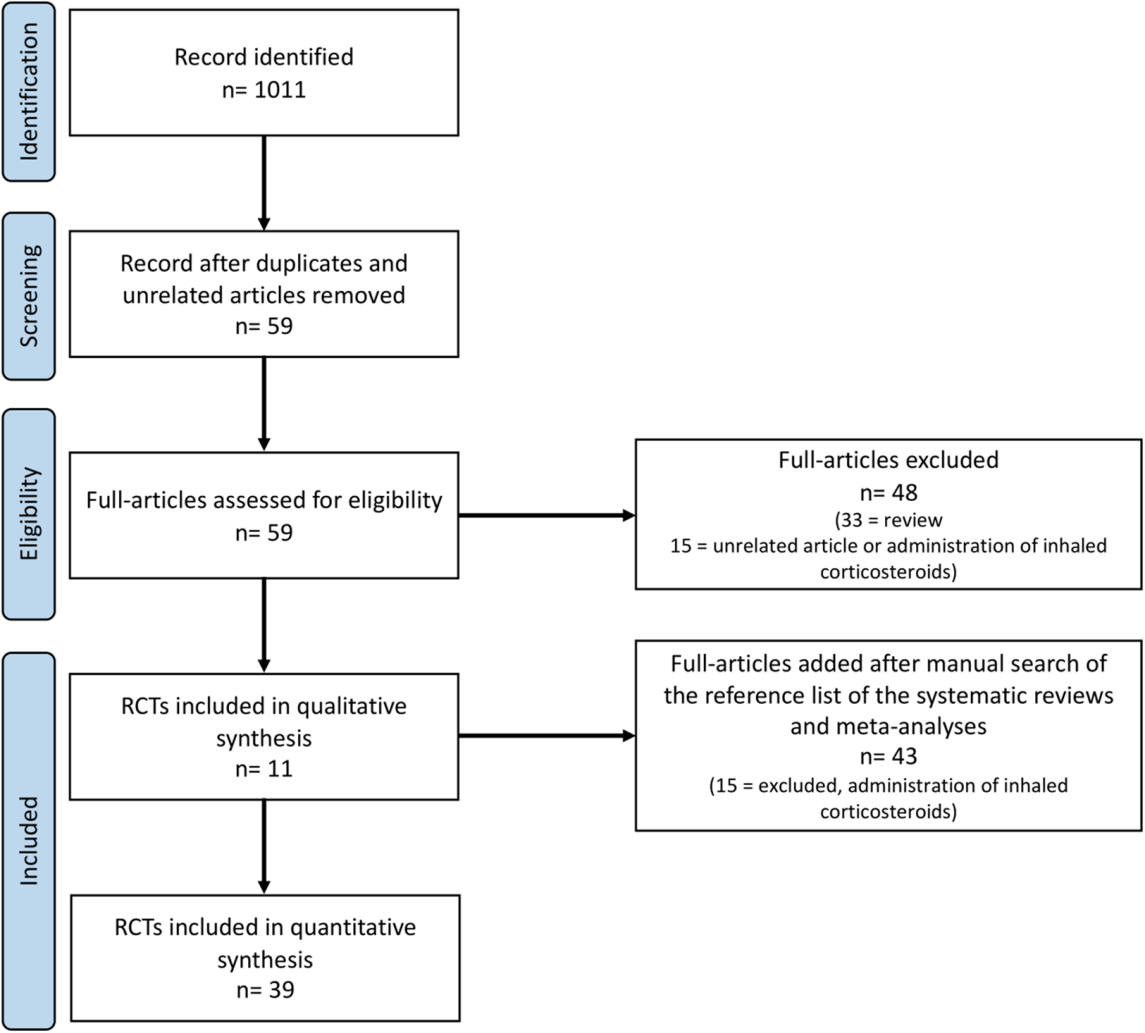
PRISMA flow-chart. RCTs, randomized controlled trials.

During the research process using the mesh term described in the Materials and methods section, we found 1,011 articles, and 11 RCTs were selected in the first qualitative synthesis after the screening process (Figure 1). After a manual search of the reference list of the systematic reviews and meta-analyses analyzed in the previous stages, we added 28 RCTs, and 39 RCTs were analyzed in the final step of this systematic review (Figure 1). Data extracted are summarized in Tables 1–6 (16–54).

**Table 1:** Randomized controlled trials comparing hydrocortisone vs. placebo.

Reference (authors, year)	Population (*n*)	Inclusion criteria	Exclusion criteria	Doses of steroids	Start at	Extubation (days of life)	Duration of therapy by protocol (days)	More than one cycle of steroids	Administration of other steroids
Halbmeijer (16)	Intervention: 182 Control: 190	GA <30 weeks and/or BW <1,250 g, ventilator dependent in the II week of life	No consent	Tapered dosing scheme of 22 days with a cumulative dose of 72.5 mg/kg	7–14 days after birth	Intervention: 9 Control: 15	22	ND	Placebo group (56.8%) treated with open-label hydrocortisone (excluded for sensitivity analyses)
Watterberg (17)	Intervention: 398 Control: 402	GA <30 weeks intubated for at least 7 days at 14–28 days	Congenital anomalies, indomethacin or ibuprofen treatment within 48 h before trial entry, and previous systemic glucocorticoid treatment	4 mg/kg/day tapered over a period of 10 days (4 mg/kg/day for 2 days, 2 mg/kg/day for 3 days, 1 mg/kg/day for 3 days, and 0.5 mg/kg/day for 2 days)	14–28 postnatal days	ND	10	ND	Open-label dexamethasone was administered to the hydrocortisone group (39.7%) and placebo group (42.1%)
Onland (18)	Intervention: 181 Control: 90	GA <30 weeks and/or BW 1,250 g, ventilator dependent between 7 and 14 days of life and at high risk of BPD	Chromosomal defects, congenital malformations, previous corticosteroids for improving lung function in the I week of life	5 mg/kg/day in 4 doses for 7 days, followed by 3.75 mg/kg/day in 3 doses for 5 days, subsequently lowering the frequency by 1 dose every 5 days (cumulative dose of 72.5 mg/kg for 22 days)	7–14 days after birth	ND	22	ND	The rate of open-label glucocorticoid use in the hydrocortisone group was 28.2% and 56.8% in the placebo
Baud (19)	Intervention: 194 Control: 185	GA <27 weeks	PROM before 22 weeks of GA, SGA, perinatal asphyxia, congenital malformations, or chromosomal aberrations	1 mg/kg/day into two doses for 7 days, followed by 0.5 mg/kg/day for 3 days	First 10 days of life	ND	10	ND	ND
Baud (20)	Intervention: 255 Control: 266	GA <27 weeks	PROM before 22 weeks of GA, SGA, perinatal asphyxia, congenital malformations, or chromosomal aberrations	1 mg/kg/day into two doses for 7 days, followed by 0.5 mg/kg/day for 3 days	First 10 days of life	ND	10	ND	ND
Parikh (21)	Intervention: 29 Control: 29	BW <1,000 g 10-days of life ventilated	<23 weeks, previously treated with corticosteroids, or indomethacin, presumed sepsis or NEC, congenital malformation	3 mg/kg/day for the first 4 days, 2 mg/kg/day for 2 days, 1 mg/kg/day for 1 day; total of 17 mg/kg over 7 days	10 and 21 postnatal days	ND	7	ND	ND
Parikh (22)	Intervention: 31 Control: 33	BW <1,000 g 10-days of life ventilated	<23 weeks, previously treated with corticosteroids, or indomethacin, presumed sepsis or NEC, congenital malformation	3 mg/kg/day for the first 4 days, 2 mg/kg/day for 2 days, 1 mg/kg/day for 1 day; total of 17 mg/kg over 7 days	10 and 21 postnatal days	ND	7	ND	Six infants in the hydrocortisone (19%) and seven in the placebo (21%) received postnatal corticosteroids after study drug completion (*P* = 0.85). Six infants (2 in the hydrocortisone group and 4 in the placebo group) received dexamethasone for ventilator-dependent BPD and five infants (3 in the hydrocortisone group and 2 in the placebo group) received hydrocortisone for suspected adrenal insufficiency
Peltoniemi (23)	Intervention: 21 Control: 19	GA <30 and BW <1,250 g and the requirement for mechanical ventilation	Malformations or early death	2.0 mg/kg for 2 days, 1.5 mg/kg for 2 days, and 0.75 mg/kg for 6 days, started before 36 h of life	Before the age of 36 h	ND	10	ND	ND
Watterberg (24)	Intervention: 126 Control: 126	BW <1,000 g and intubated	Congenital anomaly, congenital sepsis, postnatal glucocorticoid treatment other than hydrocortisone, triplet or higher-order multiple gestation	1 mg/kg/day divided twice daily for 12 days, followed by 0.5 mg/kg/day for 3 days	12 and 48 h of life	ND	15	ND	ND
Bonsante (25)	Intervention: 25 Control: 25	BW <1,500 g, GA <30 weeks, need of mechanical ventilation after surfactant administration	Malformations, perinatal asphyxia, death within 12 h after recruitment, and use of steroids for any clinical reason before and during the 12 days of treatment	0.5 mg/kg/12 h for 9 days, 0.5 mg/kg/day for 3 days	Before 48 h of life	ND	12	ND	ND
Ng (26)	Intervention: 24 Control: 24	GA <32 weeks, BW <1,500 g, hypotension medically treated	Congenital or chromosomal abnormalities, postnatal systemic or inhaled corticosteroids treatment of severe lung disease before receiving the trial drug, proven systemic infection or NEC, or underwent major surgery	1 mg/kg per dose every 8 h for 5 days	Within the first 7 days of life	ND	5	ND	ND
Peltoniemi (27)	Intervention: 25 Control: 26	GA <30 and BW <1,250 g and the requirement for mechanical ventilation	Malformations or suspected chromosomal abnormalities	2.0 mg/kg for 2 days, 1.5 mg/kg for 2 days and 0.75 mg/kg for 6 days, started before 36 h of life	Before the age of 36 h	ND	10	ND	ND
Efird (28)	Intervention: 16 Control: 18	GA <29 weeks and BW <1,000 g	Malformations and chromosomal abnormalities	1 mg/kg every 12 h for 2 days, followed by 0.3 mg/kg every 12 h for 3 days	First days of life	ND	5	ND	ND
Watterberg (29)	Intervention: 179 Control: 178	BW <1,000 g and intubated	Congenital anomaly, congenital sepsis, postnatal glucocorticoid treatment other than hydrocortisone, triplet or higher-order multiple gestation	1 mg/kg/day divided twice daily for 12 days, followed by 0.5 mg/kg/day for 3 days	12 and 48 h of life	ND	15	ND	ND
Watterberg (30)	Intervention: 20 Control: 20	Appropriate for GA, BW <1,000 g and ventilated mechanically	Maternal diabetes, congenital sepsis, and SGA	1.0 mg/kg/day every 12 h, for 12 days: 9 days at 1.0 mg/kg/day, and a 3-day taper at a reduced dose of 0.5 mg/kg/day	Before 48 h of life	ND	12	ND	ND

ND, not declared; GA, gestational age; SGA, small of gestational age; BW, birth weight; PROM, premature rupture of membranes.

**Table 2:** Randomized controlled trials comparing dexamethasone vs. placebo.

Reference (authors, year)	Population (*n*)	Inclusion criteria	Exclusion criteria	Doses of steroids	Start at	Extubation (days of life)	Duration of therapy by protocol (days)	More than one cycle of steroids	Administration of other steroids
Doyle (31)	Intervention: 29 Control: 26	GA <28 weeks or BW <1,000 g, ventilator dependent in the I week of life	Congenital defects, chromosomal anomalies	0.89 mg/kg over 10 days	After 7 days of life	ND	10	ND	ND
Doyle (32)	Intervention: 35 Control: 35	GA <28 weeks or BW <1,000 g, ventilator dependent in the I week of life	Congenital defects, chromosomal anomalies	0.89 mg/kg over 10 days	After 7 days of life	Intervention: 14 Control: 21	10	Treatment with second course intervention: 10 Control: 10	A small proportion of infants in each group had been exposed to short-course, low-dose corticosteroids before trial entry, for purposes of blood pressure control
Yeh (33)	Intervention: 72 Control: 74	BW <2,000g, severe radiographic respiratory distress syndrome requiring mechanical ventilation within in the first 6 h of life	Absence of prenatal infection, congenital anomalies, and lethal cardiopulmonary status	Two doses/day, days 1 through 7, 0.25 mg/kg/dose; days 8 through 14, 0.12 mg/kg/dose; days 15 through 21, 0.05 mg/kg/dose; and days 22 through 28, 0.02 mg/kg/dose	Within 12 h after birth	ND	28	ND	ND
Walther (34)	Intervention: 19 Control: 17	GA ≤32 weeks and RDS required mechanical ventilation	Sepsis or other infection, congenital heart disease, systemic hypertension, unstable clinical status, multiple congenital anomalies	At day 7–14. 14 days-treatment (0.2 mg/kg/day start, max 1.9 mg/kg cumulative)	7–14 after birth	ND	14	ND	ND
Romagnoli (35)	Intervention: 15 Control: 15	Oxygen and ventilator dependent on the 10th day of life and at high risk of chronic lung disease	No consent	0.5 mg/kg/day for the first 6 days, 0.25 mg/kg/day for the next 6 days, and 0.125 mg/kg/day for the last 2 days of treatment (total dose 4.75 mg/kg)	At 10 days of life	NE	14	ND	Five infants in each group received two doses of 0.5 mg/kg dexamethasone to facilitate weaning from mechanical ventilation after the first month of life
Romagnoli (36)	Intervention: 25 Control: 25	BW <1,250 g, GA <32 weeks, ventilator and oxygen-dependent at 72 h of life and at high risk of chronic lung disease	Prenatal infections, congenital malformations, and evidence of sepsis	From the 4th day of life for 7 days: 0.5 mg/kg/day for the first 3 days, 0.25 mg/kg/day for the next 3 days, and 0.125 mg/kg/day on day 7	At 4th days of life	ND	7	ND	ND
O'Shea (37)	Intervention: 50 Control: 45	BW <1,501 g, age between 15 and 25 days, <10% decrease in ventilator settings for previous 24 h and FiO2 >0.3, no signs of sepsis and echocardiogram indicating the absence of a patent ductus arteriosus	no consent	0.25 mg/kg twice a day for 3 days, then 0.15 mg/kg twice a day for 3 days, then a 10% reduction in the dose every 3 days until the dose of 0.1 mg/kg was reached on day 34. After 3 days on this dose, 0.1 mg/kg qod was given until 42 days after entry.	Between 15 and 25 days of life	NE	42	ND	ND
Kothadia (38)	Intervention: 57 Control: 61	BW <1,501 g, age between 15 and 25 days, <10% decrease in ventilator settings for previous 24 h and FiO2 >0.3, no clinical signs of sepsis and echocardiogram indicating the absence of a patent ductus arteriosus	Congenital malformation, congenital viral infection, or mother with positive serologic testing for hepatitis B or HIV	0.25 mg/kg twice a day for 3 days, then 0.15 mg/kg twice a day for 3 days, then a 10% reduction in the dose every 3 days until the dose of 0.1 mg/kg was reached on day 34. After 3 days on this dose, 0.1 mg/kg qod was given until 42 days after entry	Between 15 and 25 days of life	NE	42	ND	ND
Lin (39)	Intervention: 20 Control: 20	BW <2,000g, radiography respiratory distress syndrome, ventilated at 6 h of life	Prenatal infection, congenital anomalies, and lethal cardiopulmonary status shortly after birth	Days 1–7: 0.25 mg/kg/dose; days 8–14: 0.12 mg/kg/dose; days 15–21: 0.05 mg/kg/dose; days 22–28: 0.02 mg/kg/dose	First days of life	NE	28	ND	ND
Yeh (40)	Intervention: 132 Control: 130	BW <2,000g, severe radiographic respiratory distress syndrome requiring mechanical ventilation within the first 6 h of life	Absence of prenatal infection, congenital anomalies, and lethal cardiopulmonary status	Two doses/day, days 1 through 7, 0.25 mg/kg/dose; days 8 through 14, 0.12 mg/kg/dose; days 15 through 21, 0.05 mg/kg/dose; and days 22 through 28, 0.02 mg/kg/dose	<12 h of life	ND	28	ND	Six infants in the dexamethasone group (8%) and seven in the control group (9%) who had severe BPD required glucocorticoid therapy after the completion of the initial study. Because of the relatively short duration of therapy, these were included in the analyses as members of their initially assigned groups
Suske (41)	Intervention: 14 Control: 12	GA <34 weeks with surfactant-treated respiratory distress syndrome	Septicaemia during the I week of life, relevant cardiac anomalies, except for patent ductus arteriosus, or malformations	0.5 mg/kg IV into two fractions, for 5 days, first doses <2 h after the first surfactant dose	<2 h of life	Intervention: 6 Control: 14	5	ND	ND
Brozanski (42)	Intervention: 39 Control: 39	BW 1,500 g, ventilator support at 7 days of postnatal age	Congenital anomalies, pulmonary hypoplasia, or hemodynamic instability	Beginning at 7 days of postnatal age pulse doses of 0.25 mg/kg/dose for 3 days, repeated every 10 days until the infant reached 36 weeks postmenstrual age, or the infant no longer required ventilator support or supplemental oxygen	After 7 days of life	ND	3 days, repeated every 10 days until the infant reached 36 weeks postmenstrual age, or the infant no longer required ventilator support or supplemental oxygen	ND	ND
Durand (43)	Intervention: 23 Control: 20	BW of 501–1,500 g, GA 24–32 weeks, ventilator-dependent at 7–14 days of age despite weaning trials, ventilator rate more than 15 cydes/min, Fi02 requirement of 0.30 or more to maintain a pulse oximeter oxygen saturation of 90% or more	Documented sepsis, evidence of systemic hypertension, congenital heart disease, renal failure, grade IV intraventricular hemorrhage, and infants with multiple congenital anomalies	0.5 mg/kg/day in two divided doses for the first 3 days, 0.25 mg/kg/day for the next 3 days, and 0.1 mg/kg/day on the seventh day	7–14 days of life	ND	7	Six patients (26%) in the dexamethasone group required a second 7-day course of steroid therapy	After the study period, 13 patients in the control group were subsequently treated with dexamethasone at a later postnatal age if the clinical team felt the infant could benefit from dexamethasone therapy
Kari (44)	Intervention: 17 Control: 24	BW <1,500 g or less, GA >23 weeks, dependence on mechanical ventilation at 10 days of age, no signs of patent ductus arteriosus, sepsis, gastrointestinal bleeding, or major malformation at entry	No consent	0.5 mg/kg/day in two doses for 7 days	After 10 days of life	NE	7	ND	ND
Couser (45)	Intervention: 27 Control: 23	Required mechanical ventilation and had either traumatic or multiple intubations or if the duration of intubation was more than 14 days	Congenital anomalies, who either had previously been treated with dexamethasone for chronic lung disease or who had received pancuronium bromide therapy or other sedation 12 h before extubation	0.25 mg/kg per dose approximately 4 h before the scheduled extubation and then again, every 8 h for a total of three doses	More than 14 days of life	ND	ND	ND	ND
Cummings (46)	Intervention: 25 (42-days 13, 18-days 12) Control: 11	BW ≤1,250 g, GA ≤30 weeks, dependence on mechanical ventilation or oxygen at 2 weeks of life	Symptomatic patent ductus arteriosus, sepsis or renal failure at entry	42 days: 0.5 mg/kg/day for the first 3 days, 0.3 mg/kg/day for the next 3 days, then reduced by 10% every 3 days until a dose of 0.1 mg/kg was reached at day 34. After 3 days at this dose, the drug was given on alternate days for 1 week and discontinued 18 days: same initial dose of 0.5, but their dose then decreased more rapidly, dropping by 50% every 3 days until a dose of 0.06 mg/kg was reached at days 10. After 3 days of this dose, the drug was given on alternate days for 1 week and then discontinued. For the remaining 24 days saline placebo	After 2 weeks of life	ND	42 and 18	ND	ND

ND, not declared; GA, gestational age; BW, birth weight.

**Table 3:** Randomized controlled trials comparing different doses of dexamethasone.

Reference (authors, year)	Population (*n*)	Inclusion criteria	Exclusion criteria	Doses of steroids (intervention)	Doses of steroids (control)	Start at	Extubation (days of life)	Duration of therapy by protocol (days)	More than one cycle of steroids	Administration of other steroids
Marr (47)	Intervention: 30 Control: 29	GA ≤28 weeks radiographic findings consistent with the diagnosis of evolving BPD and ventilator support	Preexisting conditions with known increased risk for neurodevelopmental impairment. Infants with sepsis or significant patent ductus arteriosus became study-eligible if treated before the end of the enrollment window	42-day group: 0.5 mg/kg/day for the first 3 days and 0.3 mg/kg/day for the next 3 days. The dose was reduced by 10% every 3 days until a dose of 0.1 mg/kg was reached on day 34. Thereafter, this dose was maintained for 3 days, alternated daily with a saline placebo for 1 week, and then discontinued	9-day group: dexamethasone: 0.5 mg/kg/day for the first 3 days, 0.25 mg/kg/day for the next 3 days, and then 0.125 mg/kg/day for 3 days, followed by saline placebo	10–21 days of life	Intervention: 23 Control: 35	Intervention: 42 Control: 9	66% in the 9-day group qualified for only 1 course of dexamethasone, 17% received 2 courses, and 17% received all 3 courses	Two infants in each group received hydrocortisone for the treatment of refractory hypotension prior to study enrollment. One infant in the 42-day group developed hypertension and required a single dexamethasone dose reduction
Odd (48)	Intervention: 16 Control: 17	BW ≤1,250 g, ventilated between 1 and 3 weeks of life	Anomalies and surgical problems	0.5 mg/kg/day for 3 days, 0.3 mg/kg/day for 3 days, then a dose decreasing by 10% every 3 days to 0.1 mg/kg/day over a further 30 days, then 0.1 mg/kg on alternate days for one further week	0.5 mg/kg/day for 3 days, 0.3 mg/kg/day for 3 days, 0.1 mg/kg/day for 3 days, then 0.1 mg/kg every 72 h until the infant was extubated and required a FiO2 <0.25 for three doses (9 days)	After 7 days of life	Intervention: 17 Control: 22	42	ND	ND
Malloy (49)	Intervention: 8 Control: 8	GA <34 weeks BW ≤1,500 g ventilator dependent	Congenital and chromosomal anomalies, necrotizing enterocolitis, or culture-proven sepsis, who had already received any corticosteroid treatment	0.5 mg/kg/day for 3 days followed by 0.3 mg/kg/day for 4 days, every 12 h	0.08 mg/kg/day for 7 days, every 12 h	<28 days of age	ND	Intervention: 16 Control: 13	ND	ND
McEvoy (50)	Intervention: 29 Control: 33	BW <1,500 g, GA <32 weeks, ventilator dependent at 7–21 days	Congenital anomalies, documented sepsis, systemic hypertension, renal failure, and grade IV intraventricular hemorrhage	0.5 mg/kg/day for 3 days, 0.25 mg/kg/day for 3 days, and 0.1 mg/kg/day on day 7 (total dose of 2.35 mg/kg). All daily doses administered every 12 h	0.2 mg/kg/day for 3 days and 0.1 mg/kg/day for 4 days (total dose of 1 mg/kg). All daily doses administered every 12 h	7–14 days of age	ND	7	ND	Three infants in the high dose and one in the low dose had one dose held: 2, secondary to bright red blood in the orogastric tube; 1, due to increased systolic blood pressure; 1, inadvertently not given
Durand (51)	Intervention: 23 Control: 24	BW <1,500 g, GA <32 weeks, ventilator dependent at 7–21 days	Documented sepsis, systemic hypertension, renal failure, grade IV intraventricular hemorrhage, and congenital anomalies or chromosomal abnormalities	0.5 mg/kg/day for 3 days, 0.25 mg/kg/day for 3 days, and 0.1 mg/kg/day on day 7 (total dose of 2.35 mg/kg). All daily doses administered every 12 h	0.2 mg/kg/day for 3 days and 0.1 mg/kg/day for 4 days (total dose of 1 mg/kg). All daily doses administered every 12 h	7–14 days of age	ND	7	ND	After the study period, 5 patients (22%) in the high dose and 7 (29%) in the low dose were treated with dexamethasone at a later postnatal age, at the discretion of the neonatologist
Armstrong (52)	Intervention: 31 Control: 33	BW <1,250 g and ventilated at >15 cycles/minute at 7 days of life	Major congenital malformation or who were ventilated for surgical reasons	(Long group) 0.5 mg/kg/day for 3 days, reduced to 0.3 mg/kg/day for 3 days and thereafter reduced by 10% every 3 days to wean over 42 days	(Pulse group) 0.5 mg/kg/day for 3 days, repeated every 10 days until infants no longer required ventilatory support or supplemental oxygen or until 36 weeks of age	At 7 days of life	ND	ND	ND	Eleven babies received steroids outside the study protocol and were not randomized
Merz (53)	Intervention: 15 Control: 15	BW <1,250 g, GA <30 weeks, ventilator dependent at 7 days	Sepsis, congenital anomalies, suspected chromosomal abnormalities, or evidence of systemic hypertension	At 7 days of life (early treatment group). Starting dose of 0.5 mg/kg per day on the first 3 days followed by 0.3 mg/kg on days 4–6. From day 7 0.1 mg/kg, given alternatively every 2nd day from days 10 to 16	At 14 days of life same doses of the intervention group (late treatment group)	From day 7 or day 14 of life	Intervention: 14 Control: 24	10–16	ND	ND
Bloomfield (54)	Intervention: 21 Control: 19	BW <1,250 G and ventilated At >15 cycles/min at 7 days of life	Congenital malformation or who were ventilated for surgical reasons	(Long group) 0.5 Mg/Kg/Day for 3 days, reduced to 0.3 Mg/Kg/Day for 3 days and thereafter reduced by 10% every 3 days to wean over 42 days	(Pulse group) 0.5 Mg/Kg/Day for 3 days, repeated every 10 days until infants no longer required ventilatory support or supplemental oxygen or until 36 weeks of age	At 7 days of life	Intervention: 42 Control: 34	ND	ND	ND

ND, not declared; GA, gestational age; BW, birth weight; BPD, bronchopulmonary dysplasia.

**Table 4:** Randomized controlled trials comparing hydrocortisone vs. placebo.

Reference (authors, year)	Extubation	Reintubation	BPD	Others respiratory outcome	Systemic hypertension	HG	Sepsis/infection	PDA	IVH	NEC	ROP	Long-term NDV	Long-term growth
Halbmeijer (16)	**+**	NE	NE	**+**	−	−	NE	NE	NE	NE	NE	NE	NE
Watterberg (17)	**+**	NE	=	=	−	=	=	=	=	=	=	At 22–26 months =	At 22–26 months =
Onland (18)	**+**	NE	=	ND	=	−	**+** ^a^	=	=	=	NE	NE	NE
Baud (19)	ND	NE	**+** ^b^	=	ND	ND	−^b^	**+** ^b^	=	=	ND	At 2 years: **+**^b^	NE
Baud (20)	**+**	NE	=	=	=	=	−^b^	**+**	=	=	=	NE	NE
Parikh (21)	NE	NE	=	NE	NE	NE	NE	NE	=^c^	NE	NE	18 months of corrected age =	18 months of corrected age =
Parikh (22)	=	NE	=	=	=	=	=	NE	=^c^	=	NE	NE	NE
Peltoniemi (23)	NE	NE	=	NE	NE	NE	NE	**+**	=	−^d^	=	At 2 years: =	At 2 years: =
Watterberg (24)	NE	NE	=	NE	NE	NE	NE	NE	=	=^d^	=	Adjusted age 20 months **+**	Adjusted age 20 months =
Bonsante (25)	NE	NE	=	**+**	=	=	=	=	=	=	=	NE	NE
Ng (26)	NE	NE	=	**+**	=	=	=	NE	=	=	=	NE	NE
Peltoniemi (27)	=	NE	=	**+**	=	=	=	**+**	=	−^d^	=	NE	NE
Efird (28)	NE	NE	=	=	=	=	=	=	=	=	=	NE	NE
Watterberg (29)	NE	NE	=	=	=	=	=	=	=	−^d^	=	NE	NE
Watterberg (30)	ND	ND	**+**	**+**	=	=	=	=	=	=	=	NE	NE

**+**, Better outcome for intervention group (steroids administration); −, worse outcome for intervention group (steroids administration); =, no differences between intervention and control groups; NE, not evaluated; ND, not declared; BPD, bronchopulmonary dysplasia; HG, hyperglycemia; PDA, patent ductus arteriosus; IVH, intraventricular hemorrhage; NEC, necrotizing enterocolitis; ROP, retinopathy of prematurity; NDV, neurodevelopment; GA, gestational age; BW, birth weight.

^a^ Pneumonia.

^b^ For those born at 24–25 weeks of gestational age.

^c^ White matter injury.

^d^ Gastrointestinal perforation.

**Table 5:** Outcomes of randomized controlled trials comparing dexamethasone vs. placebo.

Reference (authors, year)	Extubation	Reintubation	BPD	Others respiratory outcome	Systemic hypertension	HG	Sepsis/infection	PDA	IVH	NEC	ROP	Long-term NDV	Long-term growth
Doyle (31)	NE	NE	NE	=	NE	NE	NE	NE	NE	NE	NE	At 2 years =	At 2 years =
Doyle (32)	**+**	=	=	**+**	=	=	=	=	=	=	=	NE	NE
Yeh (33)	NE	NE	**+**	**+**	NE	NE	=	NE	=	NE	=	At 8 years −	At 8 years −
Walther (34)	**+**	ND	=	=	=	−	=	NE	=	=	NE	NE	NE
Romagnoli (35)	NE	NE	**+**	NE	NE	NE	=	=	=	=	NE	24 and 36 months of life =	24 and 36 months of life =
Romagnoli (36)	**+**	ND	**+**	**+**	=	−	=	=	=	=	=	NE	NE
O'Shea (37)	NE	NE	**+**	NE	NE	NE	NE	NE	−^a^	NE	NE	At 1 year −^b^	At 1 years =
Kothadia (38)	ND	ND	**+**	**+**	=	=	=	NE	−^a^	=	=	NE	NE
Lin, 1999 (39)	=	ND	**+**	**+**	−	−	=	NE	=	NE	NE	NE	NE
Yeh (40)	**+**	ND	**+**	**+**	−	−	−	**+**	=	=	=	NE	NE
Suske (41)	**+**	ND	**+**	**+**	=	=	=	=	=	=	**+**	NE	NE
Brozanski (42)	ND	ND	**+**	**+**	NE	=	=	=	**+**	=	=	NE	NE
Durand (43)	**+**	ND	**+**	**+**	=	=	=	=	=	=	=	NE	NE
Kari (44)	ND	ND	NE	**+**	−	=	=	=	=	=	NE	NE	NE
Couser (45)	ND	**+**	ND	**+**	=	=	NE	NE	NE	NE	NE	NE	NE
Cummings (46)	**+**	=	NE	**+**	=	=	=	NE	NE	NE	=	6 And 15 Months **+**	6 And 15 Months =

^a^ Abnormal cranial ultrasound.

^b^ Cerebral palsy.

**+**, Better outcome for intervention group (steroids administration); −, worse outcome for intervention group (steroids administration); =, no differences between intervention and control groups; NE, not evaluated; ND, not declared; BPD, bronchopulmonary dysplasia; HG, hyperglycemia; PDA, patent ductus arteriosus; IVH, intraventricular hemorrhage; NEC, necrotizing enterocolitis; ROP, retinopathy of prematurity; NDV, neurodevelopment; GA, gestational age; BW, birth weight.

**Table 6:** Randomized controlled trials comparing different doses of dexamethasone.

Reference (authors, year)	Extubation	Reintubation	BPD	Others respiratory outcome	Systemic hypertension	HG	Sepsis/infection	PDA	IVH	NEC	ROP	Long-term NDV	Long-term growth
Marr (47)	**+**	**+**	=	=	=	=	=	ND	=	=	=	7 years **+**	7 years: =
Odd (48)	=	ND	NE	=	=	=	=	NE	=	NE	NE	9 and 18 months of postnatal age =	NE
Malloy (49)	=	ND	=	=	−	=	NE	NE	=	=	=	At 1 years −	NE
McEvoy (50)	NE	NE	=	**+**	=	=	=	NE	=	=	=	At 1 years =	NE
Durand (51)	NE	NE	=	**+**	=	=	=	NE	=	=	=	NE	NE
Armstrong (52)	ND	ND	**+**	=	=	NE	NE	NE	=	NE	NE	18 months of =	18 months of =
Merz (53)	**+**	ND	=	**+**	=	=	=	NE	NE	=	=	NE	NE
Bloomfield (54)	NE	NE	**+**	**+**	=	=	=	NE	NE	=	=	NE	NE

**+**, Better outcome for intervention group (steroids administration); −, Worse outcome for intervention group (steroids administration); =, no differences between intervention and control groups; NE, not evaluated; ND, not declared; BPD, bronchopulmonary dysplasia; HG, hyperglycemia; PDA, patent ductus arteriosus; IVH, intraventricular hemorrhage; NEC, necrotizing enterocolitis; ROP, retinopathy of prematurity; NDV, neurodevelopment; GA, gestational age; BW, birth weight.

### Primary outcome: long-term NDV effects and growth

**Figure 2:**
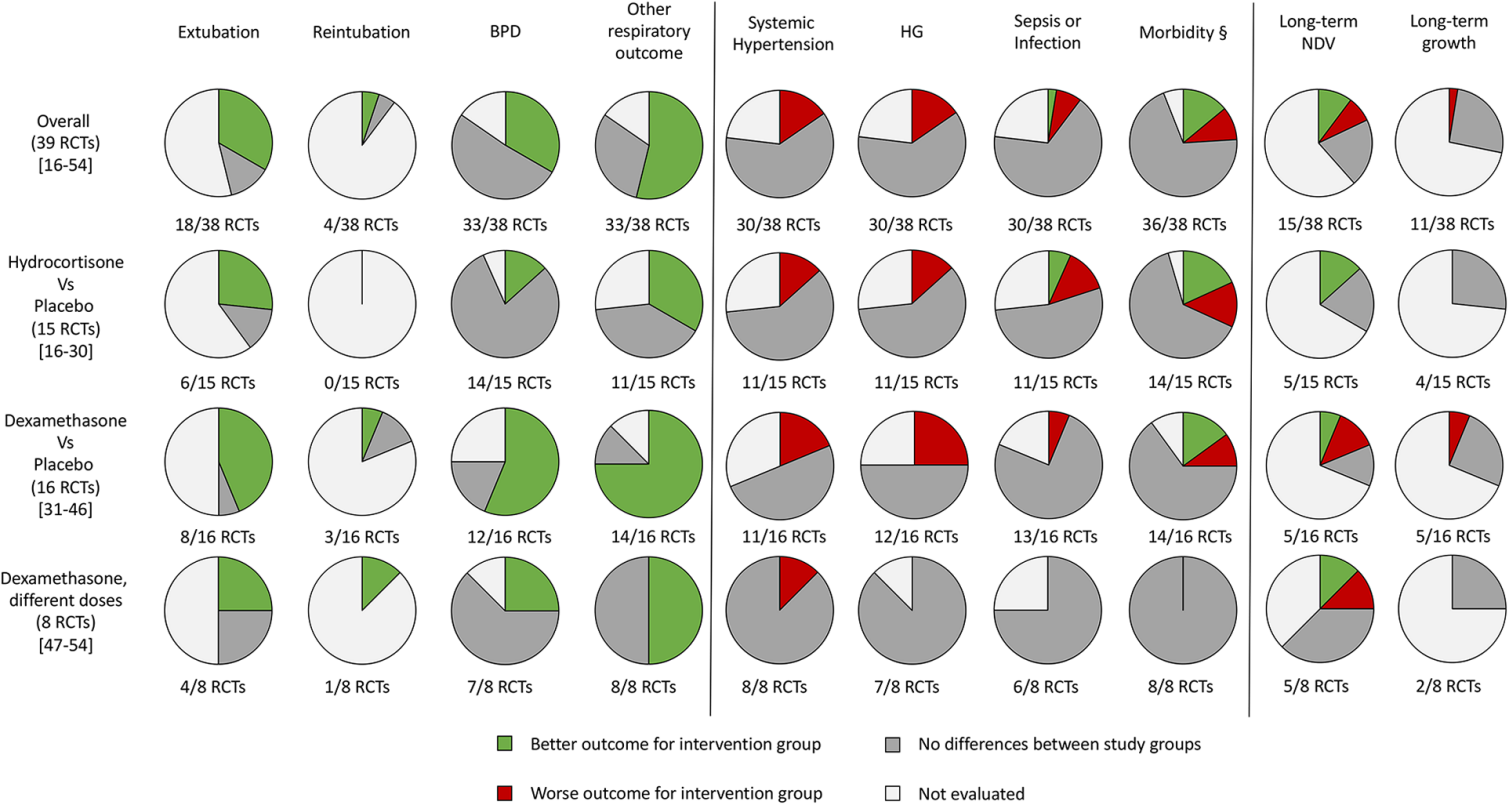
Graphical representation of the randomized controlled trials that evaluated the outcomes of the systematic review. RCTs, randomized controlled trials; BPD, bronchopulmonary dysplasia; HG, hyperglycemia; NDV, neurodevelopment; §, at least one between patent ductus arteriosus, intraventricular hemorrhage, necrotizing enterocolitis (or gastrointestinal perforation) and retinopathy of prematurity.

A graphical representation of the percentage of the studies evaluating the outcomes of interest for this study is shown in Figure 2 (16–54).

Of the 38 RCTs, 15 performed a follow-up program considering NDV, growth, or both (17, 19, 21, 23, 24, 31, 33, 35, 37, 46–50, 52).

Eight studies did not find statistically significant long-term neurological differences (17, 21, 23, 31, 35, 48, 50, 52). Malloy et al. (49) found an increased risk of NDV delay, and O'Shea et al. (37) showed an increased risk of cerebral palsy at 1 year in the groups receiving dexamethasone (different doses vs. placebo, respectively). A long-term follow-up study performed by Yeh et al. (33) showed worse effects on neuromotor and cognitive function at school age in newborns who received dexamethasone compared to the placebo group. Watterberg et al. (24) (hydrocortisone vs. placebo), Cummings et al. (46) (dexamethasone vs. placebo), and Marr et al. (47) (different doses of dexamethasone) found an improved long-term NDV. Baud et al. (19) found better NDV outcomes at 2 years in newborns who received hydrocortisone if they were born at 24–25 weeks of GA, while no statistical difference considering those born at 26–27 weeks of GA. All the RCTs that considered growth parameters did not find differences between the two groups (17, 23, 24, 31, 35, 37, 46, 47, 52). Only Yeh et al. (33) found that newborns who received dexamethasone were significantly shorter than the controls and had a significantly smaller head circumference, evaluated at school age. However, the long-term NDV and growth outcomes evaluated in these 15 RCTs were measured at different time points (Tables 4–6).

To better characterize the effects of corticosteroids on long-term neurological outcomes, we performed also a sub-analysis separating the studies for early (before 7 days of life) and late (after 7 days of life) administration. The graphical representation of this sub-analysis is reported in Supplementary Figure S1. We excluded the studies of Baud et al. (19, 20) for this analysis, because the starting age of the intervention was before 10 days of life, and based on the definition of early and late administration of corticosteroids considered, we were not able to add these studies in the analysis.

### Secondary outcome: respiratory outcome, metabolic effects, and morbidity during hospital stay

Despite most of the studies not finding differences for all the outcomes evaluated for both hydrocortisone and dexamethasone, systemic hypertension and HG appear to be the most frequent side effects, especially for dexamethasone compared with hydrocortisone (Figure 2). Both have an important effect on respiratory outcome and time to extubation (Figure 2). The reintubation rate has been rarely evaluated (Figure 2).

We found that 18 studies evaluated the early extubation rate (16–18, 20, 22, 27, 32, 34, 36, 39–41, 43, 46–49, 53). Five of 18 RCTs found no differences between intervention and control groups (2 hydrocortisone vs. placebo, 1 dexamethasone vs. placebo, and 2 with different doses of dexamethasone) (22, 27, 39, 48, 49), while 13 found an early time of extubation in newborns who received system corticosteroids (4 hydrocortisone vs. placebo, 7 dexamethasone vs. placebo, and 2 with different doses of dexamethasone) (16–18, 20, 32, 34, 36, 40, 41, 43, 46, 47, 53). A total of 21RCTs did not evaluate this outcome or declare the rate of extubation in relation to the administration of systemic corticosteroids (19, 21, 23–26, 28–31, 35, 37, 38, 42, 44, 45, 50–52, 54). Only four studies declared the rate of reintubation, specifically two RCTs found a better reintubation rate for newborns treated with systemic corticosteroids (one dexamethasone vs. placebo and one with different doses of dexamethasone) (45, 47), and two studies found no differences for newborns treated with dexamethasone (32, 36). A total of 33 studies evaluated the rate of BPD (17–30, 32–43, 47, 49–54). Thirteen of 33 found a better outcome (2 hydrocortisone vs. placebo, 9 dexamethasone vs. placebo, and 2 with different doses of dexamethasone) (19, 30, 35–40, 42, 43, 52, 54), while 20 studies did not find differences (17, 18, 20–29, 32, 34, 41, 47, 49–51, 53). Baud et al. found a better outcome for babies born at 24–25 weeks of GA whereas no differences for those born at 26–27 weeks of GA (19, 20). No studies found an increased rate of BPD between the groups. Thirty-three of 39 studies evaluated the pulmonary function (16, 17, 19, 20, 22, 25–34, 36, 38–54). In addition, 21 of 33 RCTs found an improved respiratory outcome for intervention groups (5 hydrocortisone vs. placebo, 12 dexamethasone vs. placebo, and 4 with different doses of dexamethasone) (16, 25–27, 30, 32, 33, 36, 38–46, 50, 51, 53, 54). Twelve did not find a difference (17, 19, 20, 22, 28, 29, 31, 34, 47–49, 52), whereas six did not evaluate this outcome (18, 21, 23, 24, 35, 37).

Systemic hypertension was evaluated in 30/39 studies (16–18, 20, 21, 25–30, 32, 34, 36, 38–41, 43–54). Six of 30 found an increased rate of systemic hypertension in the intervention group (2 hydrocortisone vs. placebo, 3 dexamethasone vs. placebo, and 1 with different doses of dexamethasone) (16, 17, 39, 40, 44, 49). Nine did not evaluate this outcome (19, 21, 23, 24, 31, 33, 35, 37, 42), while the rest of the 24 RCTs found no differences between the study groups (9 hydrocortisone vs. placebo, 8 dexamethasone vs. placebo and 7 with different doses of dexamethasone) (18, 20, 22, 25–30, 32, 34, 36, 38, 41, 43, 45–48, 50–54). As with systemic hypertension, a quasi-totality of the study (30/39) evaluated the outcome HG (16–18, 20, 22, 25–30, 32, 34, 36, 38–51, 53, 54). Six of 30 found an increased rate of HG in intervention groups (2 hydrocortisone vs. placebo and 4 dexamethasone vs. placebo) (16, 18, 34, 35, 39, 40) compared with control, while 24 did not find differences (17, 20, 22, 25–30, 32, 38, 41–51, 53, 54). Brozanski et al. (42) found no differences in the rate of HG, but newborns in the intervention group (dexamethasone vs. placebo) received more statistically significant insulin therapy. Ng et al. (26) and Couser et al. (45) found an increased incidence of glycosuria in the intervention group (hydrocortisone vs. placebo and dexamethasone vs. placebo, respectively), despite no difference in HG rate. Thirty studies considered the risk of sepsis or other infections (17–20, 22, 25–30, 32–36, 38–44, 46–48, 50, 51, 53, 54). Despite the almost total of the study (26/30) did not find differences (17, 22, 25–30, 32–36, 38, 39, 41–44, 46–48, 50, 51, 53, 54), the two RCTs of Baud et al. found a statistically increased risk for babies that received hydrocortisone, born at 24–25 weeks of GA (19, 20). Onland et al. found a statistically reduced incidence of pneumonia for the group of babies that received hydrocortisone (18). Only one study that compared dexamethasone vs. placebo, found an increased risk for the intervention group (40). For the outcome PDA, we considered the incidence and the treatment (medical or surgery ligation) as PDA. Eighteen RCTs considered this outcome (17–20, 23, 25, 27–30, 32, 35, 36, 40–44). Five/18 found a better outcome for treated newborns (4 hydrocortisone vs. placebo and 1 dexamethasone vs. placebo) (19, 20, 23, 27, 40). Specifically, Baud et al. found a better outcome for babies born at 24–25 weeks of GA, treated with hydrocortisone (19, 20). Thirteen RCTs did not find differences between treated and not treated newborns (17, 18, 25, 28–30, 32, 35, 36, 41–44), whereas no studies found a worse outcome. None of the studies that compared different doses of dexamethasone evaluated this outcome. IVH was considered in 33 of the 39 studies (17–30, 32, 34–44, 47–52). Only O'Shea et al. (37) and Kothadia et al. (38) demonstrated an increased risk of abnormal cranial ultrasound for neonates that received corticosteroids, whereas Brozanski et al. (42) showed a reduced risk of IVH in intervention groups. All three studies compared dexamethasone vs. placebo (37, 38, 42). The rest of the 29 studies found no statistical differences for IVH or white matter injury (17–26, 28–30, 32, 34–36, 39–41, 43, 44, 47–52). Of the 29 studies that evaluated NEC, the studies of Watterberg et al. and Peltoniemi et al. found an increased risk of gastrointestinal perforation in neonates that received corticosteroids (specifically hydrocortisone) (23, 27, 29). The other 26 RCTs did not find statistically significant differences between the two groups for NEC or gastrointestinal perforation (17–20, 22, 24–26, 28, 30, 32, 34–36, 38, 40–44, 47, 49–51, 53, 54). The risk of ROP was evaluated by 25/39 RCTs (17, 20, 23–30, 32, 33, 36, 38, 40–43, 46, 47, 49–51, 53, 54). Twenty-four did not find any statistical differences between the two trial groups (17, 20, 23–30, 32, 33, 36, 38, 40, 42, 43, 46, 47, 49–51, 53, 54), but only Suske et al. (41) demonstrated a reduced risk of ROP for newborns of the intervention groups, who were administered dexamethasone.

### Risk of bias

**Figure 3:**
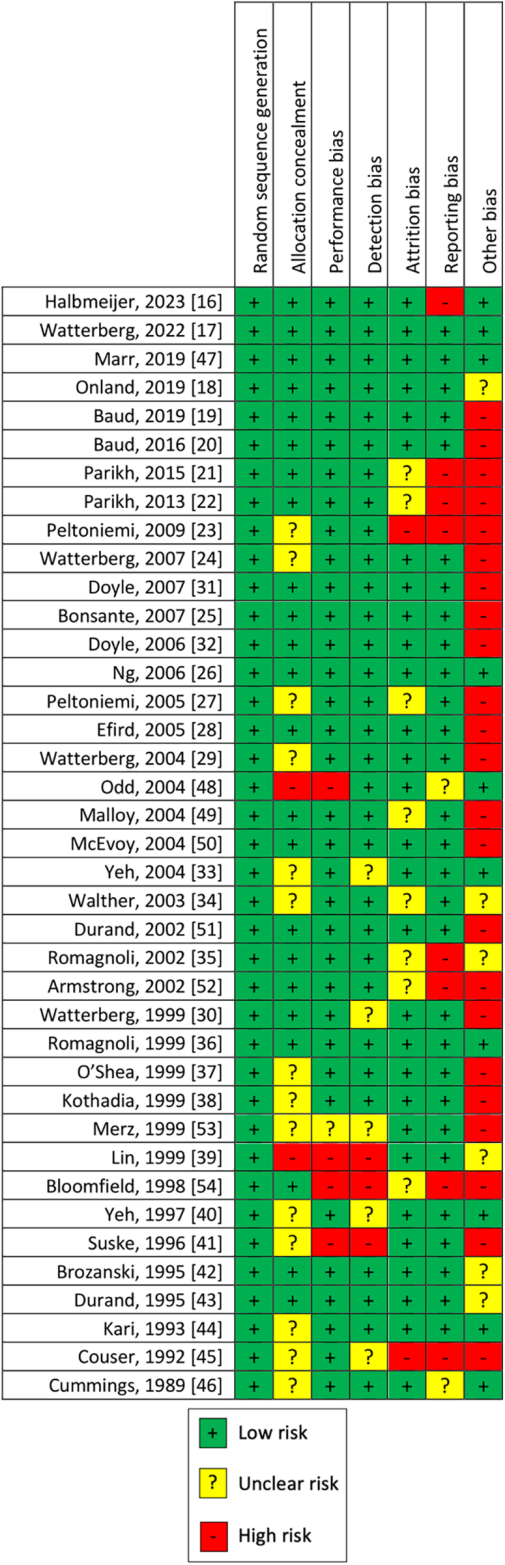
Risk of bias of the evaluated randomized controlled trials.

The quality of the studies was assessed by risks of bias, as shown in Figure 3. We judged the risk of selection bias as low in all uncontrolled studies, unclear for 14, and high for 2 RCTs. Performance bias and detection bias were high for four and three studies, respectively, and unclear for one and five, respectively. The rest of the 34 and 31 RCTs were judged as low risk of performance and detection bias, respectively. Attrition bias was judged as low for 29 studies, high for 2, and unclear for 8 RCTs. Considering our outcome, reporting bias was judged high for 8, low for 29, and unclear for the remaining 2 RCTs. Other sources of bias were not evaluable for 6 controlled trials, while was high for 23 studies and low only for 10.

## Discussion

Despite many studies exploring the effects of corticosteroid administration in postnatal life for preterm newborns, the optimal modality of administration remains to be defined. The high heterogeneity of the included RCTs regarding dosages, timing of administration, and outcome measures, discourages the mathematical analysis of the data (55). A recent meta-analysis and network meta-analysis included only one study for a therapeutic regiment or more than one study but with different timing of administration and were focused primarily on BPD (11). In this systematic review, we evaluated the efficacy of steroid therapy on other respiratory outcomes, including extubation rate, reintubation, BPD, and related side effects, considering separately hydrocortisone, dexamethasone, and different doses of dexamethasone. Despite the analyzed studies suggesting an overall favorable effect of steroids on respiratory outcomes, a well-designed large RCT is urgently needed to establish the optimal indication and modalities of administration. Among the steroids used for preterm newborns, early and high doses of dexamethasone have a better impact on the respiratory outcome, while hydrocortisone is related to fewer side effects such as systematic hypertension or HG.

### Long-term NDV effects and growth

A major limitation in the analysis of the studies including the use of steroids in the neonatal period is represented by the lack of data for long-term outcomes. Only a few studies evaluated neurological and growth outcomes of preterm newborns receiving steroids in early life (17, 19, 21, 23, 24, 31, 35, 37, 46–50, 52). Only one-third of the trials on hydrocortisone evaluated the long-term effects on NDV up to 2 years of life (17, 19, 21, 23, 24). In these studies, neurological outcomes seem to be not influenced by the use of hydrocortisone. Two RCTs found an improvement in neurological outcomes, particularly in ELGAN and newborns with extremely low birth weight (19, 24). Almost half of studies on dexamethasone evaluated neurological outcomes up to 1–2 years of life (31, 35, 37, 46–50, 52). The results of these studies are controversial. If the majority of the RCTs demonstrated that there is no influence of dexamethasone on long-term NDV (31, 35, 48, 50, 52), O'Shea et al. (37) reported an increased rate of cerebral palsy at 1 year for newborns treated with dexamethasone (42 days of therapy, starting doses at 0.25 mg/kg twice a day for 3 days) compared with placebo. Malloy et al. (49) found a worse NDV long-term outcome for newborns in the high doses of dexamethasone groups (0.5 mg/kg/day for 3 days followed by 0.3 mg/kg/day for 4 days every 12 h vs. 0.08 mg/kg/day for 7 days every 12 h). In addition, Yeh et al. (33) showed that children treated with two doses per day of dexamethasone for 28 days (0.25 mg/kg/dose up to 7 days and then the dose was tapered) had significantly poorer motor skills, motor coordination, visual–motor integration, and significantly lower full IQ, verbal IQ, and performance IQ scores. The frequency of clinically significant disabilities was significantly higher among children in the dexamethasone group compared with controls (39% vs. 22%, *p* value 0.04) (33). However, O'Shea et al. did not power the study for long-term NDV and selection bias because differential survival rates across the two study groups could explain the greater risk of cerebral palsy among the intervention group; Malloy et al. performed a trial with a very low sample size (8 vs. 8). On the other hand, Cummings et al. and Marr et al. found a better long-term neurological outcome for babies treated with dexamethasone (42 days of therapy, starting dose at 0.5 mg/kg/day for the first 3 days, 0.3 mg/kg/day for the next 3 days, and then reduced by 10% every 3 days until a dose of 0.1 mg/kg was reached at day 34) (46, 47). They enrolled newborns born ≤30 weeks of GA (and ≤1,250 g) and ≤27 weeks of GA, respectively. Thus, it could be possible that ELGAN should benefit more than other newborns from steroid treatment. In addition, Marr et al. performed a study with a long time follow-up evaluation of 7 years. Most of the studies did not consider long-term growth as an outcome. The available studies suggest a low impact of early steroid treatment and long-term growth (17, 23, 24, 31, 35, 37, 46, 47, 52). Only Yeh et al. demonstrated that dexamethasone could negatively influence height and head circumference, evaluated at school age (33).

### Respiratory outcome, metabolic effects, and morbidity during hospital stay

Postnatal steroid treatment is beneficial for respiratory outcomes, such as extubation, reduced risk of BPD or duration of invasive or non-invasive MV, supplemental oxygen therapy, FiO2, and/or other specific ventilatory/respiratory data. Dexamethasone has a better impact compared to hydrocortisone, but the optimal therapeutic regiment remains to be defined. For the reintubation rate outcome, a conclusion cannot be made, since it has been evaluated only in a few, unpowered studies (32, 45–47). In addition, in all these studies, dexamethasone was used as an intervention, while none of them considered hydrocortisone.

Our analysis showed that BPD is the most studied respiratory outcome. The majority of the studies demonstrated that dexamethasone had a positive effect on the BPD rate, whereas hydrocortisone appears to not improve this outcome. The study with a lower risk of bias showed that the therapeutic regiment of dexamethasone in early life (0.5 mg/kg/day for the first 3 days, 0.25 mg/kg/day for the next 3 days, and 0.125 mg/kg/day on the day 7) had the best impact on BPD (36).

Steroids work as anti-inflammatory agents, which can explain their rationale in the prevention of BPD. One of the main risk factors for developing BPD is prolonged oxygen exposure and MV, which induce a pulmonary local inflammatory response (4, 5). Postnatal corticosteroids decrease inflammation and edema, improving gas exchange and lung protective mechanisms (56, 57).

To the best of our knowledge, there are no RCTs that evaluated the long-term pulmonary outcome of preterm newborns treated with corticosteroids in neonatal life. On the other hand, studies aimed at investigating long-term respiratory function in preterm babies were mainly focused on BPD and did not independently evaluate the role of postnatal steroid treatment on the final outcome (58).

Not all studies evaluated side effects associated with the use of steroids in preterm newborns.

Despite the majority of the RCTs found no difference between the study groups (17, 18, 20, 22, 25–30, 32, 34, 36, 38, 41–54), some studies suggested an increased risk of systemic hypertension and/or HG.

Systemic hypertension was analyzed in the majority of the studies included in this systematic review. Few studies (∼16%) reported an increased risk of hypertension in newborns treated with steroids. The administration of dexamethasone has been associated with systemic hypertension more frequently compared to hydrocortisone. Treatment regimens of more than 0.25 mg/kg of dexamethasone, especially for more than 10 days, appear to increase the risk of systemic hypertension.

HG is an independent risk factor for mortality and NDV delay in newborns (59, 60); thus, all efforts should be made to reduce the risk of this condition. A higher number of studies on dexamethasone reported an increased risk of HG compared with studies on hydrocortisone. However, all the studies that compared two different doses or timing for dexamethasone administration found no difference for HG (47–54). When steroid treatment is needed, neonatologists should minimize the other conditions that induce an increased risk of HG (such as nutrition) (60, 61), or they should improve continuous glucose monitoring to maintain euglycemia (62).

Sepsis remains the major cause of morbidity and mortality in preterm newborns (63). Our analysis showed a relevant increased risk of sepsis in newborns treated with steroids, more for hydrocortisone compared with dexamethasone. However, a recent study demonstrated a reduced risk of pneumonia (18) in newborns treated with hydrocortisone, probably due to an improvement in respiratory outcome and a reduced time of invasive ventilation support.

Data regarding the relationship between steroid administration and morbidity conditions are controversial. Most studies suggested no relation (about 69%), while others demonstrated an increased rate of prematurity-related morbidities (about 14%); finally, about 10% suggested a better outcome. We speculated that this result depends on the different modalities of administration of corticosteroids, different therapeutic regiments, and different morbidity definitions of the studies. Based on our findings, more than 42 days of dexamethasone therapy, more than 1 mg/kg starting dose, or more than 10 days of hydrocortisone therapy might increase the risk of morbidity.

We speculate that part of the reduction in morbidity might be related to the improvement in PDA closure, associated with steroid use. Some studies demonstrated that steroids (especially hydrocortisone) could improve the PDA outcome, reducing the need for medical or surgical treatment (19, 20, 23, 27, 40). Several reasons may explain this effect: (1) *in vitro* studies demonstrated that hydrocortisone treatment decreases the sensitivity of the ductus arteriosus to the relaxing action of prostaglandin E2, which explains the beneficial effects *in vivo* of steroids (64, 65), and (2) the relationship between PDA and BPD, especially for ELGAN, has been demonstrated (66). Despite this topic is not well evaluated, it could be possible that the effects of steroid administration on PDA also improve BPD. However, future studies should evaluate this aspect.

Brozanski et al. (42) found a reduced risk of IVH for preterm receiving steroids. The authors administered a pulse dose of 0.25 mg/kg/dose of dexamethasone to newborns at 7 days of life for 3 days, repeated every 10 days until 36 weeks of postmenstrual age or up to weaning of ventilation/oxygen support. In addition, they affirmed that the decreased rate of IVH in the intervention (pulse) group could be associated with a better stabilization of capillary membranes or alteration of cerebral blood flow by corticosteroids or by an improvement in the ventilatory status of the infants (42). However, two RCTs underline the effects of steroids on early brain damage (37, 38). They administered 0.25 mg/kg twice a day for 3 days, 0.15 mg/kg twice a day for other 3 days, and then a 10% reduction in the dose every 3 days until the dose of 0.1 mg/kg was reached on day 34 and after 3 days on this dose. However, all three studies are not powered for this outcome. Thus, considering the different therapeutic regiments and this limitation, further studies are needed to clarify this aspect.

Concerns remain regarding the risk of spontaneous gastrointestinal perforations in newborns treated with hydrocortisone (23, 27, 29). In particular, Peltomieni et al. (23, 27) administered hydrocortisone 2.0 mg/kg for 2 days, 1.5 mg/kg for 2 days, and 0.75 mg/kg for 6 days, started before 36 h of life (duration therapy 10 days), and Wetterberg et al. (29) 1 mg/kg/day divided twice daily for 12 days starting at randomization (12–48 h of life), followed by 0.5 mg/kg/day for 3 days (duration therapy 15 days). In both studies, The authors stopped the studies because of the higher rate of spontaneous gastrointestinal perforation, limiting the power of the studies (23, 27, 29). Studies with similar treatment did not find differences. In addition, the majority of the analyzed RCTs did not find differences in terms of NEC or spontaneous gastrointestinal perforations between treated and placebo groups (17–20, 22, 24–26, 28, 30, 32, 34–36, 38, 40–44, 47, 49–51, 53, 54).

### Risk of bias

Given that some studies with concerns about the overall risk of bias have been included, our results need to be confirmed by further RCTs with a low risk of bias. Blinding bias was low for most of the studies analyzed. Attrition and reporting bias were judged low for most of the studies reviewed. Major concerns are about the risk of other bias (early interruption of the trial due to data-dependent process or bias related to the specific study design), which was judged high for 23 of 38 studies. In addition, the studies presented a high heterogeneity of inclusion criteria (e.g., GA and/or BW) and intervention (different doses, timing of administration, and duration therapy), which could have influenced the results. We included only RCTs, despite some of these enrolling a small number of patients with low power of the study. Some of the studies included adopted a non-optimal blinding method or were unclear.

### Strengths and limitations

Our results should be interpreted considering the limitations of the studies analyzed and of the review process. First, we decided not to perform a meta-analysis because of the extreme variability in methodology, modality of administration of steroids, and outcome of the studies (55). We systematically collected evidence and after a deep evaluation and discussion between the authors, we decided not to make a meta-analysis considering the wide differences in methodology used in different studies included in this manuscript. In particular, the studies vary regarding inclusion and exclusion criteria, enrollment, dose of treatment, starting days and duration of steroids, type of steroids administered, timing of follow-up, and assessment scales (Tables 1–3). The data deriving from current evidence, including meta-analyses, are inconclusive on the long-term effects either to exclude completely that there may be consequences on the central nervous system. Thus, we believe that steroids should be used in trial settings and to collect data in large databases to verify the consequences of this therapy.

We believe that there are no minimal criteria to perform a meta-analysis and that conclusions deriving from published meta-analyses were not supported by robust statistical data. Our data might contribute to better define the modality of steroid therapy and the target population to reduce the risk of brain damage. Whether meta-analyses suggested deleterious effects of steroids on NDV, our study demonstrated that further well-designed studies are needed to reach conclusions regarding the relationship between steroid treatment in preterm newborns and long-term NDV.

We synthesized the results of different studies on NDV. However, the long-term outcomes were not analyzed at the same time point and with different NDV assessment scales. We selected articles published in the English language; thus, it is possible that some gray literature has not been analyzed. In addition, studies showing positive results have a greater likelihood of being published. Finally, in some studies, other medications were administered, based on the clinical conditions of patients, in some studies treatment was interrupted prematurely, and others were not powered for the long-term outcomes.

## Conclusion

Postnatal administration of systemic corticosteroids is an important tool for neonatologists to improve respiratory outcomes. Based on published RCTs, dexamethasone appears to be more effective than hydrocortisone for extubation, prevention of BPD, and improvement of respiratory outcomes. However, considering the deleterious effects such as HG, caution should be made during administration of dexamethasone. In addition, long-term effects on NDV and growth remain undefined. Considering that data deriving from current evidence, including meta-analyses, are inconclusive on the long-term effects to exclude completely that there may be consequences on the central nervous system, further studies are advocated to define the optimal therapeutic regiment, to improve the positive effects and reduce the side effects of steroid administration in preterm newborns.

## Data Availability

The original contributions presented in the study are included in the article/**Supplementary Material**, further inquiries can be directed to the corresponding author.

## Supplementary material

SUPPLEMENTARY FIGURE S1

Graphical representation of the randomized controlled trials that evaluated neurological outcomes in relation to early (before 7 days of life) or late (after 7 days of life) administration of steroids. RCTs, randomized controlled trials; *the studies of Baud et al. were excluded (19, 20).
