# Supplementary figures and images for: Outcomes of postnatal systemic corticosteroids administration in ventilated preterm newborns: a systematic review of randomized controlled trials
Source: Front Pediatr. 2024 Feb 14;12:1344337. doi: 10.3389/fped.2024.1344337 (PMC10899705; doi:10.3389/fped.2024.1344337)

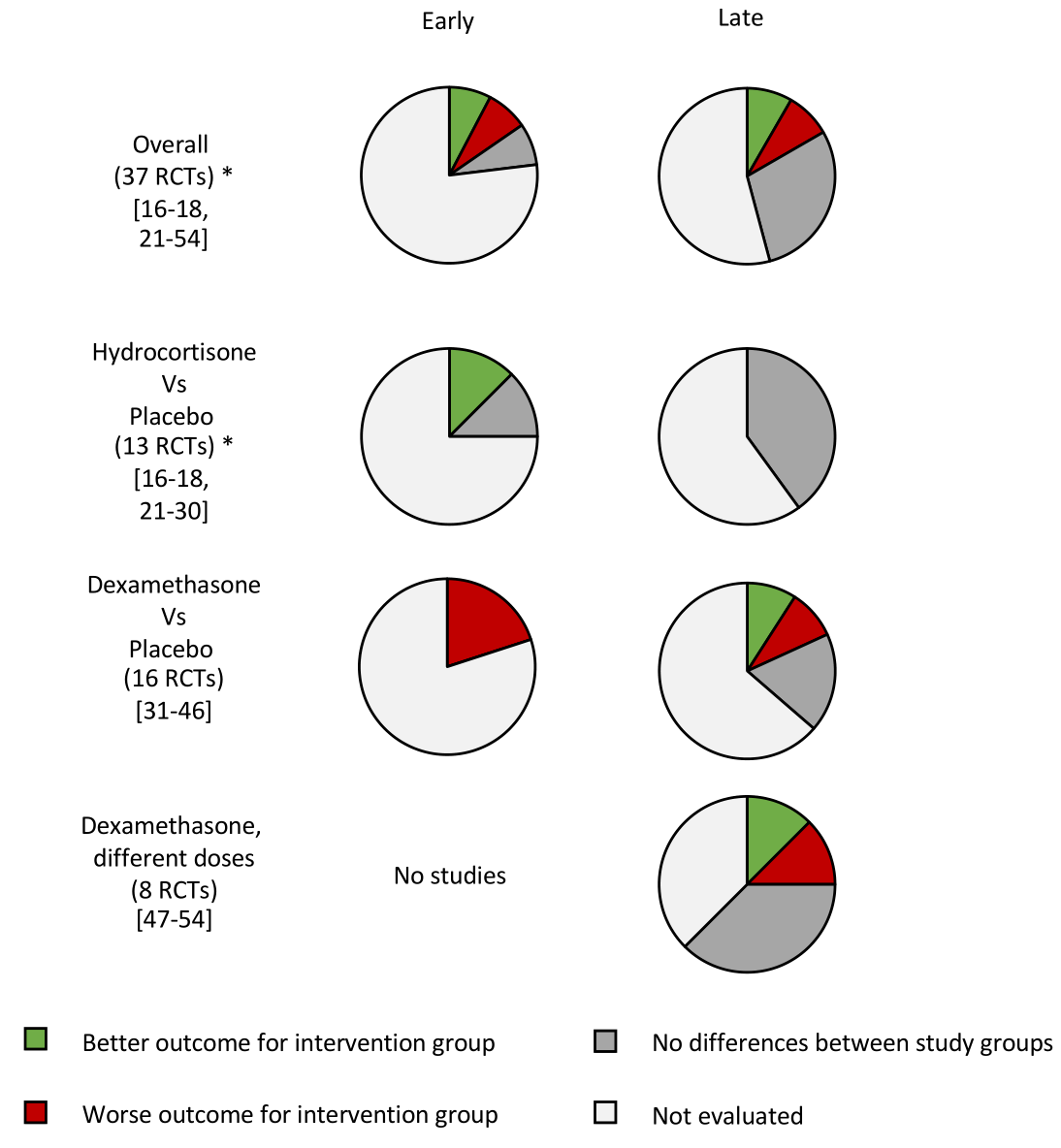

Supplement: Supplementary file 1 [file Image1.tiff]
